# Transvaginal mesh in Australia: An analysis of news media reporting from 1996 to 2021

**DOI:** 10.1111/hex.13734

**Published:** 2023-02-22

**Authors:** Mina Motamedi, Stacy M. Carter, Chris Degeling

**Affiliations:** ^1^ Australian Centre for Health Engagement Evidence and Values (ACHEEV), School of Health and Society University of Wollongong Wollongong Australia

**Keywords:** class action, epistemic injustice, innovative surgery, media analysis, Senate Inquiry, transvaginal mesh

## Abstract

**Introduction:**

Transvaginal mesh (mesh) surgeries have been used to treat stress urinary incontinence (incontinence) and/or pelvic organ prolapse (prolapse). In Australia, as in many other countries, the harms caused by mesh eventually prompted individual and collective attempts to achieve redress. The rise of mesh surgery as a procedure, the experience of mesh‐affected women and the formal inquiries and legal actions that followed all occurred in social, cultural and discursive contexts. One strategy to understand these contexts is to track how the mesh and key actors in the mesh stories have been portrayed in mass media sources. We conducted a media analysis of the most highly read Australian newspapers and online news media platforms, focusing on how mesh and the interaction of stakeholders in mesh stories were presented to the Australian public.

**Method:**

We searched systematically in the top 10 most‐read print and online media outlets in Australia. We included all articles that mentioned mesh, from the date of first use of mesh in Australia to the date of our final search (1996–2021).

**Result:**

After early scant media reporting focusing on the benefits of mesh procedures, major Australian medicolegal processes created a hook to shift reporting about mesh. The news media then played a significant role in redressing women's experienced epistemic injustice, including by amplifying previously ignored evidence of harm. This created an opportunity for previously unreported suffering to be revealed to powerful actors, in settings beyond the immediate control and epistemic authority of healthcare stakeholders, validating women's testimony and creating new hermeneutic resources for understanding mesh. Over time, media reports show healthcare stakeholders responding sympathetically to these new understandings in public discourse, contrasting with their statements in earlier media coverage.

**Conclusion:**

We argue that mass media reporting, in synergy with medicolegal actions and the Australian Senate Inquiry, appears to have provided women with greater epistemic justice, giving their testimony privileged epistemic status such that it was considered by powerful actors. Although medical reporting is not recognised in the hierarchy of evidence embedded in the medical knowledge system, in this case, media reporting appears to have contributed to shaping medical knowledge in significant ways.

**Patient or Public Contribution:**

We used publicly available data, print and online media outlets, for our analysis. Therefore, this manuscript does not contain the direct contribution of patients, service users, caregivers, people with lived experience or members of the public.

## 1 INTRODUCTION

Transvaginal mesh (mesh) surgery was developed through collaborations between surgeons and device manufacturers, in 1996, for the treatment of stress urinary incontinence (incontinence) and/or pelvic organ prolapse (prolapse) in women.^1^, ^2^ It was aggressively promoted to practitioners and rapidly adopted in practice.^3^ Clinical trials indicated mesh was effective and provided limited information regarding adverse events.^1^, ^2^, ^3^, ^4^ In contrast, the qualitative peer‐reviewed literature reveals the suffering of mesh‐affected women, including their experience of epistemic injustice.^4^

Epistemic injustice occurs when a person's contribution to the production of knowledge is unjustly excluded or dismissed.^5^ Miranda Fricker's influential account of epistemic injustice distinguishes two forms: testimonial and hermeneutic.^5^ Testimonial injustice occurs when a person's testimony is wrongly deflated by the prejudice or judgement of another, to the degree that blocks the flow of produced knowledge.^5^ Hermeneutic injustice occurs when knowledge formation is impeded due to a structurally prejudiced social gap.^5^ In other words, hermeneutic injustice occurs when a person's lived experience and produced knowledge are unfairly dismissed due to a lack of relevant interpretive resources in their community, such that others cannot make sense of their account of this experience. Mesh‐affected women's experience of testimonial and hermeneutic epistemic injustice caused compounding medical and psychological trauma and skewed the clinical evidence base.^4^ As the public controversy surrounding the safety and efficacy of mesh increased, women globally began to share stories of post‐surgical harm and complications.^4^, ^6^

It is now clear that, while benefiting some women, mesh caused iatrogenic harm to many others.^4^ One approach to understanding the sociocultural and political context for these events is to examine mass media coverage. Newspapers and other news media can disseminate health information in ways that can affect individual health choices and public health policies.^7^, ^8^, ^9^, ^10^, ^11^, ^12^ News media (media) often introduce research findings or new technologies to their readership, as well as deliver critical public health messages.^13^ Systematic analysis of health and medical reporting can reveal the cultural and political environment in which people experience and make sense of healthcare,^8^, ^9^, ^10^, ^11^, ^13^, ^14^, ^15^, ^16^ and the way in which institutions and authorities respond to and sustain public trust in healthcare practices and systems.^17^, ^18^, ^19^, ^20^ Public and political events such as government inquiries or legal actions also use the media to communicate to the population and to potential participants.

In Australia, mesh‐associated harms eventually prompted individual and collective attempts to achieve redress. The first Australian class action was filed in 2012, and the matter was referred to the Senate Community Affairs References Committee for inquiry and report on 15 February 2017.^21^ There have been similar legal actions and institutional responses in other jurisdictions (Table 1). Over 100,000 legal actions have commenced globally against mesh manufacturers (manufacturers) and healthcare providers.^4^, ^6^

**Table 1:** Examples of similar legal actions and institutional responses in other jurisdictions.

Year	Place	Legal actions and institutional responses
2014	Scotland	Established an independent inquiry
2014	England	The National Health Service England established a Mesh Working Group
2016	Canada	The class action against manufacturers and suppliers of mesh devices in Canada reached a settlement

To our knowledge, there has been only one study of media reporting of mesh, which focused on inaccuracies in media reporting of the US Food and Drug Administration's ban on the use of mesh for prolapse repair.^22^

In this paper, we report on an analysis of Australian media reporting about mesh, for the entire period in which mesh has been in use in this jurisdiction (1996–2021). Our aims were to track how mesh and key actors in the mesh stories have been portrayed in the most highly read Australian media platforms. Our research questions focused on how information about mesh was presented, and the interaction of stakeholders in mesh stories. By examining media reporting, we then draw conclusions about public and institutional perspectives on mesh procedures in Australia, and how the issue of mesh was presented to the Australian public. Our research questions were:

1. How did reporting of mesh change over time?
2. How were the relevant conditions and treatments constructed?
3. How were women and their experiences represented?
4. How was the mesh problem constructed by different stakeholders, and what potential solutions were presented?

## 2 METHODS

Detailed search strategy and search results are provided in Supplement 1. The online databases *Factiva* and *ProQuest* were searched for English language stories on mesh published between January 1996 and November 2021. We searched in the top 10 most‐read print and online media outlets in Australia, using the rankings contained in the most recent analysis by Australian market research company, Roy Morgan.^23^, ^24^ When sources were not available in *Factiva* or *ProQuest*, we conducted a manual search of the relevant publishers' websites.

**Figure 1:**
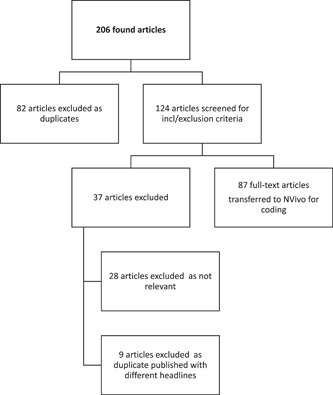
Preferred Reporting Items for Systematic Reviews and Meta‐Analyses flow diagrams.

Initial searches identified 206 articles (see Figure 1). After excluding exact duplicates, we included all articles that mentioned mesh—even in passing—as a surgical treatment for incontinence and/or prolapse in women. Full‐text reports of 124 articles were downloaded for full‐text review, of which 28 were excluded because the content was not relevant. Our final sample was comprised of 96 articles, of which 9 were duplicates published with different headlines, and syndicated to multiple state‐based publications; these were excluded.

### 2.1 Data extraction and coding

All 87 articles were transferred to NVivo for coding and analysis, which occurred in two phases. M.M. coded all articles line by line. Initial coding combined deductive codes based on earlier analysis of the qualitative literature on women's experience of mesh surgery, and inductive codes developed from this sample. The coding scheme particularly focused on the range of stakeholders and how they were represented, and on how conditions, treatments, problems and solutions were constructed. Based on this draft coding, the main code book was developed iteratively through collaboration and discussion between the three authors. M.M. then used this final codebook to recode the data set. The interpretation was developed collaboratively between the three authors, synthesising themes through discussion between all three authors, over several iterations. Any uncertainties were resolved through discussion to ensure coherence and alignment with study objectives. See Supplement 2 for characteristics of included news media articles reporting on mesh from 1996 to 2021.

## 3 RESULTS

### 3.1 How did reporting of mesh change over time?

**Figure 2:**
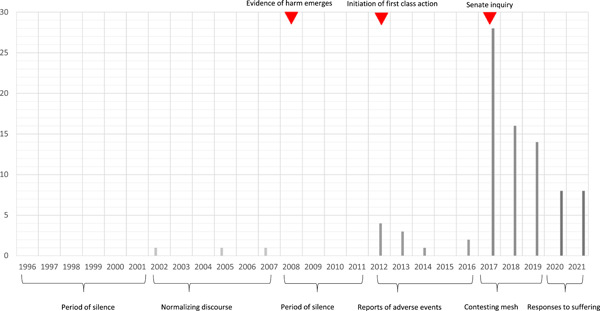
Distribution of articles over the study period.

As shown in Figure 2, the publication of articles can be organised into six time periods.

From 1996 to 2001, mesh was in use, but we found no media reporting. We identified three articles published between 2002 and 2007, that focused on a *normalising* discourse: framing incontinence and prolapse as common medical conditions reducing women's quality of life and encouraging women to seek help. Within these articles, mesh was introduced as a new, innovative, minimally invasive, highly successful, accessible treatment option for women. From 2008 to 2011, there was a second *period of silence*. Only from 2012 to 2016, in the leadup to the first Australian class action and Senate Inquiry, did *reports of adverse events* begin to draw media attention.

Between 2017 and 2019 media reports began to *contest the assumed value of mesh*—65% of included stories reported on the Senate Inquiry and the first Australian class action. Within these articles, mesh was depicted in an overwhelmingly negative way. Reports focused on women's experience of severe adverse events; the Senate Inquiry and the class action maintained newsworthiness. In the final period—2020–2021—media reports focused on institutional *responses to women's suffering*. In this period, journalists reported on stakeholders' responses to the outcomes of the Senate Inquiry and the class action; mesh‐associated harms remained central, along with women's struggles to obtain help and support, including from the legal system.

In the final two periods (2017–2021), other innovative treatments for incontinence and/or prolapse—such as transvaginal laser therapy and repair using stem cells—began to be reported. (In transvaginal laser therapy, a small attachment is reportedly inserted into the vagina for treating urethral tissue with quick laser pulses. Light emitted thermal effects reportedly stimulate and encourage the growth of new collagen and elastin. Stem cell repair reportedly involves using stem cells from a woman's uterus combined with nanobiomaterials to repair damaged tissues; it was reportedly introduced after testing in sheep but had yet to undergo clinical trials.) Notably, the tone and framing of these innovations were similar to how incontinence and/or prolapse were represented before 2016. That is, pairing an argument that incontinence and/or prolapse were medical conditions impairing quality of life and requiring treatment, with innovative treatment as a solution. Unlike the reporting in 1996–2016, these stories consistently included negative information about mesh and ended with a call for long‐term safety and efficacy research before implementation.

### 3.2 How were the relevant conditions and treatments constructed in media reporting?

Throughout the period sampled, incontinence and prolapse were constructed in media reporting as ‘silent epidemic’, stigmatised, serious and historically and unjustly trivialised conditions.^25^, ^26^, ^27^ Both conditions were ‘secret women's business’,^28^, ^29^ ‘too intimate’ to be discussed with doctors, shameful and taboo, such that women ‘suffer[ed] in silence’.^25^, ^28^, ^30^, ^31^ Reporting was strongly gendered with a minority of stories mentioning incontinence in men and children. Stories told women that these conditions were not normal,^26^, ^28^, ^30^, ^32^ and would become increasingly debilitating if left untreated.^28^, ^30^ A link to childbirth was often reported (38% of stories); often supported by women's accounts of patronising gendered exchanges with medical professionals:

you've had three kids, what do you expect?…^25^

… she had told her GP of her incontinence and met the response: ‘What are you doing with that? You haven't had children’^32^

Most often, stories emphasised that incontinence and prolapse are socially and psychologically damaging, causing social isolation and undermining the quality of life and confidence.^25^, ^26^, ^29^, ^33^, ^34^, ^35^, ^36^ Some stories adopted a traditional story arc, first building tension via the negative construction of the condition, then relieving that tension by presenting a cure.^26^, ^28^ Early stories featured non‐surgical treatments^25^, ^26^, ^32^ but also mentioned mesh as a ‘more accessible’ treatment.^26^ In early stories especially, medical professionals framed mesh positively, emphasising transformation of the field, complete cure and quality‐of‐life improvements.^26^, ^34^, ^35^, ^37^, ^38^, ^39^ Later stories were more likely to emphasise the risk of complications in all surgical procedures, the need for care, respect and consideration in treatment decision making or that mesh may be more suitable for some women than others (e.g., older women).^34^, ^35^, ^36^, ^37^, ^38^

As reporting began to focus on the Senate Inquiry and class action, two starkly contrasting constructions of mesh emerged. More than two‐thirds of media stories pitted women and their advocates (who typically used descriptors such as ‘scandal’, ‘failed’ or ‘disastrous’), against manufacturers and medical professionals (who typically used descriptors such as ‘up to 99% successful’, ‘effective and safe’ or ‘revolutionary’).

Table 2 maps the proportion of stories reporting on harm. In many stories, these harms were presented as extreme but rare events: mesh was a ‘successful simple medical procedure’ but produced ‘multiple, extreme, traumatic, incapacitating and life altering injuries’ for some, changing their lives permanently.^40^, ^41^, ^42^

**Table 2:** Reported harms.

General description of harms	*n* = 87, *n* (%)
Prevalent	32 (37)
Traumatic	33 (38)
Irreparable	15 (17)

**Specific harms**	
Pain	63 (72)
Inability to have sex	35 (40)
Infection or bleeding	34 (39)
Psychological or emotional harms	34 (39)
Internal lacerations or erosion	31 (35)
Inability to work and financial difficulty	18 (20)
Worsen incontinence	16 (18)
Inability to walk, stand upright or sit	15 (17)
Repeated surgeries	11 (12)
Mesh breaking up	7 (8)
Suicidal thoughts	6 (7)
Death	2 (3)

Later reports also revealed disagreement over mesh removal procedures, which again were constructed differently by different stakeholders.^43^ Medical professionals represented mesh removal negatively, emphasising that it was sometimes unnecessary, potentially harmful, ‘extremely difficult’, ‘distressing’ and potentially ineffective in removing symptoms.^31^, ^36^, ^41^, ^43^, ^44^, ^45^, ^46^ The procedure could take large expert teams up to a full day, require multiple operations, or prove impossible.^31^, ^36^, ^37^, ^41^, ^47^, ^48^, ^49^, ^50^ In contrast, women quoted in media stories framed removal as the only escape from a life‐destroying technology. Loss of faith in Australian surgeons drove rejection of conservative treatment recommendations: women were ‘anxious’ for or ‘adamant’ about removal, and would accept risks, travel internationally or pay large sums to obtain it.^36^, ^39^, ^43^, ^51^, ^52^, ^53^

### 3.3 How were women and their experiences represented?

Many media reports related to women's personal stories, which mirrored accounts in the qualitative literature.^4^ Some illustrative quotes are provided in Supplement 3. Information is given before mesh procedures were reportedly limited (including not being informed that mesh would be used), women's symptoms after surgery were dismissed or trivialised, compounding women's trauma and women were unaware of how to report adverse events to regulators.^27^ The Senate Inquiry and the class action were framed as an important source of validation for women, reflecting and vindicating their previously dismissed symptoms and revealing a new community of similarly affected women.^54^, ^55^

Gender appeared frequently in these reports. Occasional reports were uncritically gendered: for example, incontinence and prolapse were framed as a product of incompetent female embodiment, implying that ‘white women can't squat’ and so effectively bring these conditions upon themselves.^25^, ^29^ More commonly reports on mesh, particularly after the Senate review and the class action, including a critique of gendered power structures. Stories, for example, recounted male representatives of manufacturers using demeaning gendered images and jokes to promote mesh to predominantly male surgeons,^52^, ^56^ women being dismissed post‐surgery as hypochondriacal or hysterical,^33^, ^36^, ^39^, ^41^, ^46^, ^54^, ^55^, ^56^, ^57^ and distrust of women's testimony reportedly delaying actions in the ‘often male‐dominated arena’ of the courts.^57^, ^58^, ^59^

### 3.4 How was the mesh problem constructed by different stakeholders, and what potential solutions were presented?

**Figure 3:**
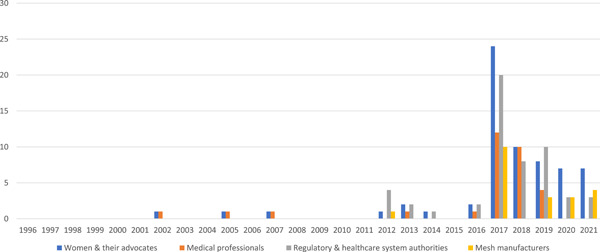
Appearance of different actors in reporting of mesh over time.

As previously noted, media reporting often constructed the issue of mesh as a battle: women and their advocates versus manufacturers, medical professionals and regulatory and healthcare system authorities. Figure 3 shows the appearance of these actors in reporting of mesh over time.

**Figure 4:**
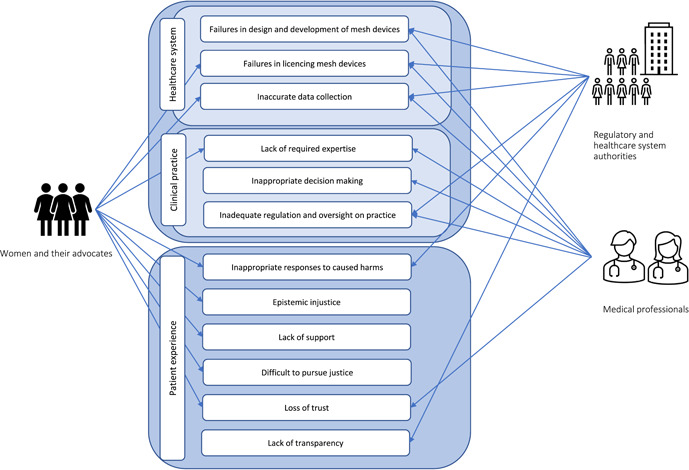
Competing constructions of the problem of mesh in media reports.

The battle between these actors was presented as one example in a series of similar incidents when women were left with no support to face devastating outcomes of cascading failures in healthcare systems.^35^, ^47^ Figure 4 depicts the competing constructions of the problem of mesh offered by different stakeholders, which we will discuss in turn below.

#### 3.4.1 Women and their advocates

Women and their advocates framed mesh as a problem in two respects: a failure of oversight, and a failure of relationality and recognition. Claims of *failure of oversight* related both to approval and monitoring, particularly with respect to the evidence base for mesh procedures. On this view, mesh device licensing was based on inadequate and unconvincing evidence. Emerging evidence of harm did not prompt needed device withdrawal, and surveillance and monitoring of mesh outcomes were poor, delaying appropriate responses.^27^, ^28^, ^35^, ^36^, ^40^, ^47^, ^51^, ^60^ Women and their advocates sometimes also suggested Australian surgeons lack necessary skills and thus caused harm.^36^ Claims of *failure of relationality and recognition* were even more prominent and drew attention to women's experience of mesh interventions and their aftermath. Accounts of women having their initial symptoms and symptoms post‐mesh removal dismissed by doctors were prominent.^27^, ^43^, ^46^, ^49^, ^50^, ^53^, ^57^, ^58^, ^59^ Regulators and health systems were also framed as slow and inadequate in providing practical support, both before and after mesh‐associated harms were recognised,^36^, ^61^ and inquiries, hearings and appeals were framed as delaying tactics by which more powerful stakeholders attempted to get ‘the answers they want’ despite women's suffering.^36^, ^57^, ^59^, ^61^ The pursuit of justice via these routes was also framed as dangerous and potentially fruitless: as the legal system is ‘expensive, intrusive and time‐consuming’, participating exposed women's vulnerability, without a large class action or a powerful supporter, women risked being framed as vexatious.^46^, ^49^, ^50^, ^57^, ^58^, ^59^ Mesh‐affected women often described a personal transition to an activist or advocate identity in which they fought for recognition for all mesh‐affected women, to not ‘let the hurt just be buried and forgotten’.^30^, ^41^, ^46^, ^47^, ^48^, ^51^, ^53^, ^58^ Women also framed their mesh experiences as undermining their ability to trust Australian medical professionals and the Australian healthcare system, preventing them from further treatment‐seeking.^43^

#### 3.4.2 Medical professionals

Medical professionals' construction of mesh as a problem had four areas of focus: device failure, clinical practice failure, regulatory and systems failure and sociocultural impacts.

Some clinicians located the problem with the devices themselves, arguing either that they had a faulty design^36^ or that they failed when ‘the device integrates within the human body’.^61^ With respect to clinical practices, some clinicians distinguished expert from ‘inexperienced surgeons’, arguing that mesh surgery was complex, and beyond the skills of ‘lesser‐trained’ surgeons, who they sought to blame for all adverse events, and who had the devices/procedures actively marketed to them.^28^, ^33^, ^34^, ^36^, ^39^ These ‘bad apples’ did not just lack clinical skill. On these accounts, they obfuscated information about their capabilities and surgical outcomes, including when seeking consent from women.^30^, ^36^, ^42^, ^58^ Alternatively, some argued that failure to recognise mesh injury was simply because ‘doctors are human and they don't all react perfectly’.^34^, ^39^

Clinical spokespeople also constructed mesh as a problem of regulatory failure, of several kinds. Licensing failures occurred when safety assessment and approval processes relied on insufficient evidence.^28^, ^36^, ^46^, ^53^, ^56^ Failure to systematically collect data on device use hampered both recognition of complications and investigation of poorly performing surgeons.^40^, ^46^, ^53^, ^56^ And lack of regulation and oversight of practice meant that the ‘bad apples’ subject to a tribunal and legal actions had years of continuous practice with insufficient regulation and oversight.^36^, ^42^, ^58^

Finally, medical professionals constructed a different kind of harm: that the legal and media environment was undermining women's trust in mesh as a treatment, and harming women by restricting their access to mesh. This included concern that helpful mesh surgeries for incontinence were being confused with harmful mesh surgeries for prolapse, and that preventing any access to mesh would be a ‘hysterical’ and ‘regrettable’ ‘over‐reaction’ as it ‘has truly been a godsend for so many women’ with incontinence.^28^, ^34^, ^38^, ^39^, ^43^ Medical professionals suggested that the majority of women who had benefited from mesh were ignored by the media, the federal court and the Senate Inquiry.^36^, ^38^ Further, medical professionals framed the adverse publicity about mesh as misleading, increasing patient anxiety and mistrust and potentially leaving women with incontinence and prolapse without effective treatment while presenting alternative treatments as useless and ineffective.^34^, ^35^, ^38^

#### 3.4.3 Regulatory and healthcare system authorities

When regulatory and healthcare system authorities appeared in media reporting, the focus was on the same four areas as medical professionals: device failure, clinical practice failure, regulatory and systems failure, and sociocultural impacts.

Device failure was constructed as the initial and leading problem in all stories.^29^, ^33^, ^43^, ^44^, ^45^, ^48^, ^49^, ^50^, ^54^, ^56^, ^62^, ^63^ Manufacturers were portrayed as responsible for this device failure due to: inadequate clinical trials; inadequate assessment of safety and efficacy of devices; concealing known and potential adverse events; minimising, downplaying or ignoring serious risks; suppressing unfavourable data; and rushing products to licensing, marketing and promotion.

Health system spokespeople also emphasised licensing failures in media reports. They often portrayed the Australian Regulator (the Therapeutic Goods Administration) as the main ‘gatekeeper’, and thereby responsible for all subsequent failures. Critics claimed the regulator had a long history of repetitive failures, leading to inquiries and calls for operational change,^36^, ^49^ and that the haphazard and voluntary process for reporting and documenting of adverse events led to underreporting and provided a false sense of safety. Consequently, data on mesh use and clinical practice was not systematically collected, therefore there was no reliable information on the number of women who had mesh surgeries, the number and types of devices used, or associated issues and complications.^40^, ^53^ The claim was that emerging problems were thus masked for years, exposing many women to mesh‐associated complications and adding to delays in responding to the caused harm.^46^, ^64^

Once issues with mesh devices began to gain media attention (2017–2019), regulatory and healthcare system authorities were increasingly represented as providing inadequate and inappropriate responses to failures, in a setting that relies on institutional and individual self‐regulation and self‐monitoring.^36^ Organisational reviews and recommendations made in response to the Senate Inquiry were depicted as being ‘complex and expensive’, adding to the regulatory confusion, and raising more questions about what needs to be done in the future.^61^ Media critiques sought to highlight that women had been already let down by previous slow responses and that a lack of transparency adds to mesh‐associated problems.^27^, ^33^, ^36^, ^42^, ^48^, ^64^

#### 3.4.4 Manufacturers

Manufacturers were not represented as speakers in the media reports sampled till the results of the class action and Senate Inquiry became public. Reporting on the federal court hearings often gave critical attention to mesh production and promotion strategies.^33^, ^34^, ^36^, ^43^, ^48^, ^49^, ^60^, ^63^ None of these articles included an interview with a manufacturer's representative, ascribed to them being unavailable or unable to comment on cases before the court.^58^ A handful of media reports described federal court proceedings where manufacturers refused liability and responsibility for caused harms, representing serious mesh‐related harms as ‘rare’ and ‘transitory’ reactions.^49^ Witnesses for manufacturers also reportedly suggested that the debilitating pain experienced by some women could be due to existing ‘competing causes’ rather than mesh products, with women's continued experience of pain after mesh removal surgery cited as evidence.^63^ Manufacturers claimed that mesh is highly successful and has helped many women: if mesh had some ‘failures [this] is simply one of its features’.^62^

Once the results of the class action and Senate Inquiry became public, manufacturers released carefully scripted statements expressing their empathy for all women who experience medical complications, while also claiming that they have ‘acted ethically and responsibly’.^45^, ^50^, ^60^ These statements described incontinence and prolapse as ‘debilitating conditions’ and emphasised the importance of making informed and shared decisions in assessing the ‘benefits and risks of surgical procedures’.^60^ The focus of these press releases was not on mesh procedures and mesh‐associated complications but rather on the more general issue of medical conditions and complications. Manufacturers thus constructed mesh devices as not particularly problematic, such that a solution was not within the scope of manufacturer's responsibilities.

#### 3.4.5 Responses and proposed solutions

From 2017, as the media raised the public profile of mesh‐related harms, news stories began to describe the systems in place in Australia to ensure patient safety and quality of care, analyse and depict responses to mesh‐associated harms, and finally describe the perspectives of different stakeholders on recommendations and proposed solutions. In these reports, medical professionals and regulatory and healthcare system authorities were depicted as responding to mesh‐caused issues while expressing their sympathy for affected women. We will outline these responses first, and then turn to proposed solutions.

##### Responses

Media reports depicted early responses from regulatory and healthcare system authorities as inappropriate, especially the inadequacies of the assessment and approval processes for mesh devices.^27^ After 2016, stories reported that regulators and the healthcare system were responding quickly to emerging global evidence on the risks and safety of mesh devices, through continuous improvement in the regulatory system to ensure quality and patient safety standards in Australia.^36^, ^40^, ^41^, ^43^, ^44^, ^45^, ^46^, ^47^, ^49^, ^60^ In 2018, after the Senate Inquiry report was released, an announcement was made in the media that public funds were to be allocated to establish specialist clinics to assess affected women and provide them with multidisciplinary services and support.^65^

Medical professionals were portrayed as being very defensive about their role in the mesh disaster, with interviewed representatives emphasising the ‘rigorous and extensive training program[s]’ undertaken by surgeons to ensure their capability to deliver the best procedures and highest quality care to patients in Australia.^36^, ^39^, ^43^ This included claims that many skilled and qualified Australian medical professionals could perform mesh implantation and removal surgery if required.^39^, ^43^

##### Solutions

**Figure 5:**
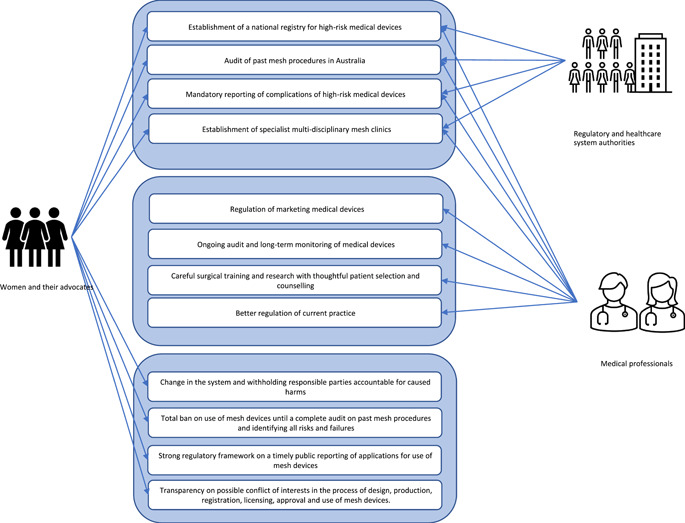
Summary of solutions attributed to different stakeholders in media reports.

Except for manufacturers, to some extent, all stakeholders were represented in media reports as supporting proposed solutions, including better transparency and monitoring regarding the use of high‐risk medical devices, such as mesh, and the establishment of specialist multidisciplinary mesh clinics.^36^, ^41^, ^42^, ^47^ However, as shown by Figure 5, in addition to these proposed solutions, affected women and their representatives used the media to advocate for further measures to redress a broader set of concerns and sociotechnical problems.

Medical professionals and regulatory and healthcare system authorities tended to advocate for solutions focused on the regulation of mesh devices and the improvement of clinical practice.^28^, ^35^, ^36^, ^43^ Solutions proposed by women and their advocates were presented in much greater detail in this media coverage, urging governments and legal authorities to hold manufactures, regulators and practitioners accountable by establishing clear responsibilities within device approval and monitoring systems.^55^, ^59^ In this reporting, mesh‐affected women strongly advocated for a total ban on the use of mesh until a complete audit of past mesh procedures was conducted.^36^, ^43^, ^48^, ^51^ Their proposed solutions also included establishing a strong regulatory framework that demanded timely reporting to the public of applications for use of mesh devices and the number of women who will be receiving them.^54^ Building on these calls for better transparency, women used media to ask for greater assurance that the normative challenges identified by the Senate Inquiry will be directly addressed—especially the need for complete transparency on possible conflict of interests for medical practitioners involved in the process of design, production, registration, licensing, approval and use of mesh devices.^36^, ^50^

## 4 DISCUSSION

We analysed all Australian media stories about mesh published from the introduction of mesh devices in the Australian healthcare system to November 2021. We identified consistently highly gendered construction of the two relevant medical conditions (incontinence and prolapse) and mesh procedures, at both an individual level and in the collective response to mesh‐caused harms. We identified six distinct periods in medical reporting on these procedures and diverse accounts of the problems and solutions associated with these procedures coming from different actors. Even though media reporting of mesh procedures changed considerably over time, the issue of mesh and diverse accounts of the problems and solutions associated with these procedures was continuously constructed as a battle between two groups of stakeholders. Women and their advocates were represented in one group, facing manufacturers, medical professionals and regulatory and healthcare system authorities.

Media reports suggested manufacturers did not recognise mesh procedures as particularly problematic. That exception noted, both the ‘women and their advocates’ group and the ‘professionals’ group were depicted as criticising failures in healthcare systems and clinical practice. After the first Australian class action and subsequent Senate Inquiry, medical professionals and regulatory and healthcare system authorities acknowledged some mesh‐associated sociocultural impacts publicly, and proposed solutions to address failures in the healthcare system and clinical practice.

Women and their advocates' quotes, unsurprisingly, focused on women's experience of mesh, which was reflected in the solutions they reportedly proposed. These included greater transparency and monitoring in the production, promotion and use of mesh devices, and further measures to address a broader set of sociotechnical problems, including a total ban on the use of mesh until a complete audit of past mesh procedures was conducted.

We argue that the media played an important role in highlighting diverse accounts of mesh—including a broader range of problems and solutions—by engaging with women's accounts of experienced epistemic injustice. We also argue that this appears to have had practical implications for the evaluation of this innovative surgical intervention. We will discuss these two issues in turn.

### 4.1 Epistemic injustice

As noted in our introduction, we have argued elsewhere that mesh‐affected women experienced significant epistemic injustice.^4^ Some news stories reported on individual women's experience of epistemic injustice—having their accounts dismissed by doctors and health systems—but these were relatively late in the reporting period. Here we extend our previous analysis by suggesting that epistemic injustice also occurred in media reporting itself.

This hinges on the timing of reporting. From the introduction of mesh in Australia, women suffered for almost 2 decades before reports of adverse events began to appear in the media. As shown in Figure 2, women's accounts received little attention until the early stages of the first Australian class action in 2012. Before this, a small number of stories consistently depicted women as having inaccurate perceptions and understandings of their own bodies and well‐being. A significant number of Australian women were experiencing adverse outcomes, but few of these women appeared in news stories. In 2017, the media interest increased considerably because of the initiation of two major Australian processes against powerful stakeholders, the first Australian class action and the Australian Senate Inquiry. It was only after these formal proceedings were launched that women's accounts were treated as newsworthy in a significant way.

Once the class action and the Senate Inquiry attracted media interest, some stories included accounts of the response harmed women had historically received. These reports suggested that before 2017, women and their experiences were frequently discounted as inconsequential, as not commercially viable for lawyers to take on in class actions, and not of interest to medical professionals, the healthcare system and regulatory authorities. It was only when women raised their concerns collectively via different routes to powerful actors that they could set the epistemic agenda around mesh, creating an opportunity and environment where they could be heard. We argue that the early period of silence on women's suffering constitutes a form of testimonial injustice in media reporting. In other words, the testimony of powerful actors was presented in early media coverage, but women's testimony was apparently not considered newsworthy.

Our previous work also highlighted that women experienced hermeneutical injustice: their families, social groups and medical professionals did not have the conceptual resources required to make sense of their experienced symptoms.^4^ This was also reflected in this media analysis. Once reporters started to take more interest in mesh‐associated harms and complications, women were able to elaborate in media stories, articulating their testimony and explaining their symptoms. In other words, greater media coverage provided the interpretive resources for a different understanding of the outcomes of mesh procedures. We would argue that it seems that the media provided women with some measure of hermeneutical justice, but only after the two formal processes in Australia. Once these newsworthy conflicts were launched, women were able to use the media to amplify their voices, creating new hermeneutical resources for others to understand their experiences. It is worth noting that there was a dialectic relationship between the legal and Senate actions and the media: the media presented the formal proceedings to the public, and the formal proceedings used the media as a channel through which to recruit testimony from women and their advocates.

Thus, this analysis suggests that the two formal proceedings in Australia, the class action and subsequent Senate Inquiry, provided a forum for recognition of women's unfavourable outcomes. This led to a major shift in the role of media publishers in this story. Initially, news accounts served largely as a thinly veiled promotion and marketing strategy for manufacturers and medical professionals. After the class action and Senate Inquiry commenced, media outlets began reporting on experiences of negative health outcomes, empowering women and amplifying their voices to compete with the influence of established and powerful institutional actors. In all earlier stories, medical professionals, and regulatory and healthcare system authorities were reported to have positive views of mesh procedures. Once the class action and Senate Inquiry launched, their official media positions changed: they qualified their continuing advocacy for mesh procedures by acknowledging experiences of adverse events, and expressed apology statements and sympathy to mesh‐affected women. At this time, media outlets also started to report the sustained advocacy of healthcare groups for the use of mesh devices, demonstrating that women's experienced health issues were constantly unjustly dismissed. This public acknowledgement was a turning point for women experiencing epistemic injustice. The media coverage of their testimony enabled them to use media reporting to amplify accounts of their experienced harms, to be recognised as knowledge‐holders regarding their own bodies, and to alleviate their experienced epistemic injustice in the healthcare system. Without this media attention, it seems possible that the class action or Senate Inquiry alone may not have achieved such a thorough public reframing of the issue of mesh. Arguably, the media coverage changed the hermeneutic resources available to the public for interpreting women's experience of mesh, and thus contributed to an increase in hermeneutic as well as testimonial justice for mesh‐affected women.

### 4.2 Evaluation of innovative surgical treatments

As noted in our introduction, clinical literature, based on case reports and clinical trials interpreted through the paradigm of evidence‐based medicine (EBM), did not capture women's experience of adverse events following mesh procedures.^4^, ^66^ Surgeons' definitions of success distorted the clinical evidence‐base and the evaluation of mesh procedures.^4^ EBM is always evolving and its principles and practice are still debated.^67^ Recent efforts to incorporate patient‐centred outcomes into the evidence base are not consistently implemented, especially in evaluations of the efficacy of surgical innovations.^66^, ^68^, ^69^, ^70^, ^71^ We argue this was certainly the case in the clinical literature on mesh procedures. This leads us to propose that media reporting of women's experiences of mesh may have played a significant role in disseminating critical information that had been epistemically marginalised in clinical contexts.

As we mentioned earlier, after the initiation of two major Australian medicolegal processes against powerful stakeholders, media reporting of mesh procedures shifted. Initially acting as a promotion and marketing strategy for mesh procedures, media reporting became a means of communicating women's experiences of negative outcomes to powerful stakeholders and decision‐makers. It seems feasible that media reporting of critiques of mesh‐caused harms may have created a new opportunity for evidence of harms to become part of the evaluative frame for these innovative procedures. After Havi Carel, this can be thought of as a process of providing patient's testimony with an epistemic privilege, that is, the privilege of being incorporated into decision making, intervention design or policy‐making.^72^ In this instance, the reported public statements of medical professionals and decision makers changed: initially, mesh was ‘highly successful’ and adverse events minimal; later, severe permanent adverse events were acknowledged publicly. The analysis of the final two time periods identified in our analysis (2017–2021) demonstrated this change, including in relation to the next new innovative treatments for incontinence and prolapse. When quoted in stories about these new innovations, rather than simply expressing enthusiasm, these groups emphasised the need for long‐term evidence of efficacy.

### 4.3 Limitations

We were unable to access rankings of media outlets by circulation for each of the years in our study. For consistency, we adopted a contemporary list of the highest circulation outlets and applied this retrospectively across all years. This may mean that we have missed a small number of reports. However, this is likely to have had a negligible effect on our analysis, as there is a high degree of syndication in the Australian media, and our sample includes longstanding media brands that have dominated the market for decades.

Our study design focused on understanding how different stakeholders and issues were represented in media discourse; a different study design could have provided more information about how media values shaped the discourse and how different media actors and mastheads participated in it.

## 5 CONCLUSION

Medical professionals and decision‐makers reportedly dismissed women's accounts of mesh‐associated harms in the early days of the procedure. After early, scant media reports focusing on the benefits of mesh procedures, major Australian medicolegal processes created a hook to shift reporting about mesh. The media then played a significant role in redressing epistemic injustice and amplified previously ignored evidence of harm. This created an opportunity for previously unreported suffering to be revealed to powerful actors, in settings beyond the immediate control and epistemic authority of healthcare stakeholders, validating women's testimony and creating new hermeneutic resources for understanding mesh.

Over time, media reports show healthcare stakeholders responding sympathetically to these new understandings, contrasting with their statements in earlier media coverage. Therefore, we argue that in this context, media reporting in combination with medicolegal action appears to have communicated new information about outcomes, shaped medical knowledge, and provided greater recognition of women's experience. This episode demonstrates the value and importance of being attentive to patient‐centred accounts of outcomes in the evaluation of innovative surgeries. It took political and legal inquiry and media reporting of experienced harms to provide women with epistemic justice and give their testimony privileged epistemic status, such that it was considered by powerful actors. Although medical reporting is not recognised in the hierarchy of evidence embedded in the medical knowledge system, in this case, media reporting appears to have contributed to shaping medical knowledge in significant ways.

## CONFLICT OF INTEREST STATEMENT

All authors declare having no conflict of interest that may have influenced this study.

## Supporting information

Supporting information.

## Data Availability

The data that support the findings of this study are available in the supplementary material of this article and were openly available in the public domain.

## References

[1] Heneghan CJ , Goldacre B , Onakpoya I , et al. Trials of transvaginal mesh devices for pelvic organ prolapse: a systematic database review of the US FDA approval process. BMJ Open. 2017;7(12):e017125. 10.1136/bmjopen-2017-017125 PMC5728256 29212782

[2] Karmakar D , Hayward L . What can we learn from the vaginal mesh story? Climacteric. 2019;22(3):277‐282. 30829077 10.1080/13697137.2019.1575355

[3] Ducey A , Donoso C , Ross S , Robert M . From anatomy to patient experience in pelvic floor surgery: mindlines, evidence, responsibility, and transvaginal mesh. Soc Sci Med. 2020;260:113151. 32738706 10.1016/j.socscimed.2020.113151

[4] Motamedi M , Carter SM , Degeling C . Women's experiences of and perspectives on transvaginal mesh surgery for stress urine incontinency and pelvic organ prolapse: a qualitative systematic review. Patient. 2021;15:157‐169. 34609727 10.1007/s40271-021-00547-7 PMC8866356

[5] Fricker M . Epistemic Justice: Power and the Ethics of Knowing. Oxford University Press; 2007.

[6] Heneghan C , Aronson JK , Goldacre B , Mahtani KR , Plüddemann A , Onakpoya I . Transvaginal mesh failure: lessons for regulation of implantable devices. BMJ. 2017;359:5515. 10.1136/bmj.j5515 29217786

[7] Bird SE . Anthropological engagement with news media: why now? Anthropology News. 2010;51(4):5‐9.

[8] Robinson C , Cutfield N , Mottershead J , et al. Media reporting of health interventions in New Zealand: a retrospective analysis. Intern Med J. 2018;48(8):924‐930. 29663620 10.1111/imj.13936

[9] Seale C . Media and Health. SAGE Publications; 2003.

[10] Dallimore DJ , McLaughlin L , Williams C , Noyes J . Media content analysis of the introduction of a “soft opt‐out” system of organ donation in Wales 2015‐17. Health Expect. 2019;22(3):485‐495. 30729625 10.1111/hex.12872 PMC6543148

[11] Maniatopoulos G , Hopkins C , Joyce TJ , Brittain K . Framing the failure of medical implants: media representations of the ASR hip replacements in the UK. Health Expect. 2019;22(3):518‐527. 30891890 10.1111/hex.12877 PMC6543155

[12] Hinchcliff R , Westbrook J , Greenfield D , Baysari M , Moldovan M , Braithwaite J . Analysis of Australian newspaper coverage of medication errors. Int J Qual Health Care. 2012;24(1):1‐8. 22117025 10.1093/intqhc/mzr067

[13] Suleski J , Ibaraki M . Scientists are talking, but mostly to each other: a quantitative analysis of research represented in mass media. Public Underst Sci. 2010;19(1):115‐125.

[14] Almomani B , Hawwa AF , Goodfellow NA , Millership JS , McElnay JC . Pharmacogenetics and the print media: what is the public told? BMC Med Genet. 2015;16(1):32. 25956914 10.1186/s12881-015-0172-3 PMC4630890

[15] Goldacre B . Preventing bad reporting on health research. BMJ. 2014;349:g7465. 10.1136/bmj.g7465 25498123

[16] Moynihan R , Bero L , Ross‐Degnan D , et al. Coverage by the news media of the benefits and risks of medications. N Engl J Med. 2000;342(22):1645‐1650. 10833211 10.1056/NEJM200006013422206

[17] Oliver JE , Lee T . Public opinion and the politics of obesity in America. J Health Polit Policy Law. 2005;30:923‐954. 16477792 10.1215/03616878-30-5-923

[18] Saguy AC , Almeling R . Fat in the fire? Science, the news media, and the “Obesity Epidemic”. Sociological Forum. 2008;23(1):53‐83.

[19] Degeling C , Rock M , Teows L . Portrayals of canine obesity in English‐language newspapers and in leading veterinary journals, 2000–2009: implications for animal welfare organizations and veterinarians as public educators. J Appl Anim Welf Sci. 2011;14(4):286‐303. 21932944 10.1080/10888705.2011.600160

[20] Lupton D . Editorial: health, illness and medicine in the media. Health. 1999;3(3):259‐262.

[21] Australian Parliament Senate Community Affairs References Committee . Number of women in Australia who have had transvaginal mesh implants and related matters. Commonwealth of Australia: Parliament House, Canberra, 1‐168. 2018. https://www.aph.gov.au/Parliamentary_Business/Committees/Senate/Community_Affairs/MeshImplants/Report

[22] Ray S , Clifton MM , Koo K . Inaccuracies in news media reporting about the 2019 US food and drug administration ban on transvaginal mesh for pelvic organ prolapse repair. Urology. 2021;150:194‐200. 32439554 10.1016/j.urology.2020.05.009

[23] Roy Morgan . Roy Morgan Research Institute. 2021. https://www.roymorgan.com/findings/8788-thinknewsbrands-total-news-readership-release-august-2021-202108200629

[24] Roy Morgan . It's official: most Australians now visit news or newspaper websites. Roy Morgan Research Institute. 2018. http://www.roymorgan.com/findings/7595-top-20-news-websites-march-2018-201805240521

[25] Baker J . Treat the leakage. *The Sydney Morning Herald*. September 15, 2005, Sect. Health & Science.

[26] Bisset K . Surgery holds answer to incontinence. *The Australian*. September 29, 2007, Sect. Review.

[27] McCarthy J . Agony of years suffering in isolation. *Sydney Morning Herald*. April 14, 2016, Sect. News.

[28] Elliott E . Suffering in silence. *Herald Sun*. June 16, 2018.

[29] Withey A . Women living with prolapse—the horrific impact of traumatic birth and exercise. *ABC Premium News*. February 28, 2018.

[30] Smail S . Women angry over side‐effects from another Johnson and Johnson product. *Australian Broadcasting Corporation*. November 25, 2013.

[31] Miles J . Women break the silence. *The Courier‐Mail*. July 2, 2017.

[32] Fannin P . Cure for a common problem mind & body—attitudes. *The Age*. January 21, 2002.

[33] Knaus C . Johnson & Johnson? Tried to prevent report about pelvic mesh devices, court hears. *The Guardian*. July 10, 2017, Sect. Australia News.

[34] Knaus C . Surgeons lacked caution in use of vaginal mesh implants, doctor admits. *The Guardian*. September 18, 2017, Sect. Society.

[35] Mutton S . ‘Not all vaginal implants are a problem’: doctor says women are refusing to seek treatment for incontinence after hundreds claimed mesh left them in pain and ruined their sex lives. *Mail Online*. June 6, 2018, Sect. Femail.

[36] Hooton A , McCarthy J . The ‘eight‐minute’ cure: how transvaginal mesh sentenced thousands of women to a life of pain. *The Sydney Morning Herald‐Online*. June 15, 2019.

[37] Dibben K . Women join national medical class action—implant caused years of pain. *The Courier‐Mail*. March 25, 2013.

[38] Overington C . Hysterical bias risks success of female surgery. *The Australian*. August 31, 2017, Sect. The Nation.

[39] Moodie C . Pelvic mesh implant patients want answers from Senate report. *ABC Premium News*. March 26, 2018.

[40] McArthur G . Help for mesh pain. *Herald‐Sun*. December 19, 2017, Sect. News.

[41] McCarthy J . Mother's surgery nightmare: pelvic mesh scandal. *Sydney Morning Herald*. December 21, 2017.

[42] Clun R . Australian doctor face of international clinical trial for controversial pelvic mesh. *The Sydney Morning Herald‐Online*. August 21, 2019.

[43] Scott S . Lamborghinis, ski trips used to market mesh implants to surgeons, documents show. *Australian Broadcasting Corporation*. August 14, 2017.

[44] Whitbourn M . Judge orders Johnson & Johnson to issue graphic warnings on pelvic mesh products. *The Sydney Morning Herald‐Online*. March 6, 2020.

[45] Whitbourn M . Court orders pharma giant to pay millions for faulty implants. *The Sydney Morning Herald*. March 6, 2021, Sect. News.

[46] Alexander L . Mesh implant dangers. *The Saturday Paper*. August 19, 2017.

[47] McCarthy J . Mum dies after pelvic mesh implant. *The Sydney Morning Herald*. December 4, 2017, Sect. News.

[48] Knaus C . Vaginal mesh risks downplayed by Johnson & Johnson, court told. *The Guardian*. July 4, 2017, Sect. Australia News.

[49] Knaus C . Australian women win landmark vaginal mesh class action against Johnson & Johnson. *The Guardian*. November 21, 2019, Sect. Australia News.

[50] Gair K . Pelvic mesh maker to pay $2.6m in damages. *The Australian—Online*. March 3, 2020, Sect. Business.

[51] Smail S . Law firm investigates more painful complications from pelvic mesh; more evidence has emerged about the potentially devastating side effects from products used to support weak pelvic floor muscles. Shine lawyers are pursuing a class action against manufacturer American Medical Systems after women allegedly suffered painful complications their products. *Australian Broadcasting Corporation*. November 18, 2014.

[52] Moore H , Rahman K . ‘I would not want my wife to undergo this procedure’: Johnson & Johnson doctor flagged concerns over ‘defective vaginal mesh’—as company faces class action. *Mail Online*. July 5, 2017, Sect. News.

[53] O'Leary C . Battle to beat pelvic mesh pain goes east. *The West Australian*. June 4, 2017, Sect. News.

[54] Scott S . Controversial vaginal mesh implants banned because of risk to prolapse patients: health authorities ban vaginal mesh implants for use in pelvic organ prolapse after the ABC reported how the devices left women in chronic, debilitating pain. *ABC Premium News*. November 29, 2017.

[55] Darvall K . ‘I could never have sex again’: how a vaginal mesh implant left a Sydney woman in horrific pain and her husband too scared to touch her—as hundreds launch class action. Mail Online. November 30, 2017, Sect. News.

[56] Knaus C . Johnson & Johnson vaginal mesh presentation featured lingerie‐clad women, court told. *The Guardian*. October 9, 2017, Sect. Australia News.

[57] Bonyhady N . Scrutiny to fall on class actions that vindicate victims. *Sydney Morning Herald*. July 13, 2020.

[58] McCarthy J . ‘I can't let the hurt be buried and forgotten’. *The Age*. March 23, 2017.

[59] Butler B . Fears a crackdown on class actions in Australia could let big businesses ‘do what they like’. *The Guardian*. July 13, 2020, Sect. Australia News.

[60] Casben L . Johnson & Johnson to pay $2.6m to women with faulty pelvic mesh implants. *ABC Premium News*. March 3, 2020.

[61] Moodie C . Vaginal mesh implants: gynaecologist urges proactive response to health concerns: a leading gynaecologist says Australia has been slow to act on the health concerns caused by pelvic mesh implants. *ABC Premium News*. July 4, 2017.

[62] Scott S . Vaginal mesh implants: Johnson and Johnson defends product in court. *ABC Premium News*. July 11, 2017.

[63] Knaus C . Johnson & Johnson doubts vaginal mesh implants cause chronic pain, court hears. *The Guardian*. July 12, 2017, Sect. Australia News.

[64] Dunlevy S . Implant horror takes toll. *The Courier‐Mail*. October 20, 2012.

[65] Clinic planned for women injured by mesh implants. *The Courier‐Mail*. Nationwide News Pty Ltd. February 11, 2018.

[66] Black N . Evidence‐based surgery: a passing fad? World J Surg. 1999;23:789‐793. 10415204 10.1007/s002689900581

[67] Lipworth W , Carter SM , Kerridge I . The “EBM movement”: where did it come from, where is it going, and why does it matter? Social Epistemol. 2008;22(4):425‐431.

[68] Rogers W , Degeling C , Townley C . Equity under the knife: justice and evidence in surgery. Bioethics. 2014;28(3):119‐126. 22681677 10.1111/j.1467-8519.2012.01980.x

[69] Rao S . Ethics on the learning curve. J Indian Assoc Pediatr Surg. 2022;27(2):191‐195. 35937115 10.4103/jiaps.JIAPS_364_20 PMC9350650

[70] Greenhalgh T , Howick J , Maskrey N . Evidence based medicine: a movement in crisis? BMJ. 2014;348:g3725. 24927763 10.1136/bmj.g3725 PMC4056639

[71] Stirrat GM . Ethics and evidence based surgery. J Med Ethics. 2004;30(2):160‐165. 10.1136/jme.2003.007054 15082810 PMC1733841

[72] Carel H , Kidd IJ . Epistemic injustice in healthcare: a philosophial analysis. Med Health Care Philos. 2014;17(4):529‐540. 24740808 10.1007/s11019-014-9560-2

