# Supplementary material for: Transvaginal mesh in Australia: An analysis of news media reporting from 1996 to 2021
Source: Health Expect. 2023 Feb 22;26(3):1189–201. doi: 10.1111/hex.13734 (PMC10154800; doi:10.1111/hex.13734)
Supplement: Supplementary file 1 — Supporting information. [file HEX-26--s001.docx]

Transvaginal mesh in Australia: an analysis of news media reporting from 1996 to 2021.

# Supplement 1

Search strategy:

"vaginal Mesh" or "transvaginal tape" or "transvaginal mesh" or ("mesh" near "vagin*") or ("tape" near "vagin*") or “tension-free vaginal tape” or “trans obturator tape”

Search results in the top 10 most-read print and online media outlets in Australia:

| Top 10 Australian most read printed media - identified by Roy Morgan in August 2021(16) | Factiva | ProQuest | Manual search |
| --- | --- | --- | --- |
| The Sydney Morning Herald | 14 | 15 |  |
| The Age | 6 | 9 |  |
| The Australian | 10 | 5 |  |
| The Daily Telegraph | 6 | 3 |  |
| The Herald Sun | 4 | 4 |  |
| The West Australian & Sunday Times | 1 | N/A |  |
| The Australian Financial Review | 2 | N/A |  |
| The Courier Mail | 6 | 9 |  |
| The Adelaide Advertiser | 1 | 3 |  |
| The Saturday Paper | N/A | N/A | 1 |
| Top 10 Australian most read online media – identified by Roy Morgan in March 2018 (17) |  |  |  |
| News.com.au | 1 | 1 |  |
| The Sydney Morning Herald | 12 | 15 |  |
| ABC news | 0 | 21 |  |
| Daily mail | 4 | N/A |  |
| The Daily Telegraph | 5 | 3 |  |
| The Age | 6 | 9 |  |
| MSN | N/A | N/A | 7 |
| The Guardian | 18 | N/A |  |
| BBC | 0 | N/A |  |
| Herald sun | 1 | 4 |  |
| Total number of articles | 97 | 101 | 8 |

Some publications appeared in both print and online versions of a publication including in: The Sydney Morning Herald, The Age, The Daily Telegraph and the Herald sun. If stories were different in print and online media, we included both, and if they were identical, we exclude one with no structured preference for online versus print versions.

Manual searches were conducted if sources were not available in Factiva or ProQuest. Also, two of the most-read online sources, The Guardian and Daily Mail, are British publications that launched Australian editions in 2013 and 2014 respectively. For this reason, we excluded articles published from 1996 to end of 2013 for The Guardian and 2014 for the Daily Mail, as these were written for British rather than Australian audiences.

Initial searches identified 206 articles. After excluding exact duplicates, we screened all 124 remaining articles by our inclusion and exclusion criteria. Our inclusion and exclusion criteria are presented in the box below:

| Inclusion criteria | exclusion criteria |
| --- | --- |
| - Mentions transvaginal/vaginal mesh or tape used as a surgical intervention for SUI and/or POP in women (even in passing) - Published Jan 1996- Nov 2021 for all outlets except The Guardian and The Daily Mail - For The Daily Mail: published 2015-2021 - For The Guardian: published 2014-2021 | - Does not mention transvaginal/vaginal mesh or tape used as a surgical intervention for SUI and/or POP in women - Published before 1996 for all outlets except The Guardian and The Daily Mail - For The Daily Mail: published before 2015 - For The Guardian: published before 2014 |

# Supplement 2 – Characteristics of included news media articles reporting on transvaginal mesh from 1996 to 2021

|  | **Author(s)** | **Year** | **Title** | **Publication** | **Reporting on women’s narratives and their experiences** | **Reporting on construction of TVM problem by** | | | |
| --- | --- | --- | --- | --- | --- | --- | --- | --- | --- |
|  |  |  |  |  |  | **Women & their advocates** | **Medical professionals** | **Regulatory & healthcare system authorities** | **TVM manufacturers** |
|  | Fannin P. | 2002 | Cure for a common problem MIND & BODY - Attitudes | The Age |  |  |  |  |  |
|  | Baker J. | 2005 | Treat the leakage | The Sydney Morning Herald |  |  |  |  |  |
|  | Bisset K | 2007 | Surgery holds answer to incontinence. | The Australian |  |  |  |  |  |
|  | Wordsworth M. | 2012 | Johnson & Johnson facing massive class action; A woman who was left in agony after surgery to repair damage caused during childbirth is now the face of what could be the largest product class action in Australian legal history | ABC Premium News |  |  |  |  |  |
|  | Wordsworth M. | 2012 | Medical giant faces history-making class action | Australian Broadcasting Corporation |  |  |  |  |  |
|  | Dunlevy S. | 2012 | Device with death High toll of implants. | Herald Sun |  |  |  |  |  |
|  | Dunlevy S. | 2012 | Implant horror takes toll | The Courier - Mail |  |  |  |  |  |
|  | Dibben K. | 2013 | Women join national medical class action - Implant caused years of pain | The Courier - Mail |  |  |  |  |  |
|  | Smail S. | 2013 | Women angry over side-effects from another Johnson and Johnson product | Australian Broadcasting Corporation |  |  |  |  |  |
|  | Smail S. | 2013 | Hundreds of women join class action against Johnson and Johnson over pelvic floor products | ABC Premium News |  |  |  |  |  |
|  | Smail S. | 2014 | Law firm investigates more painful complictions from pelvic mesh; More evidence has emerged about the potentially devastating side effects from products used to support weak pelvic floor muscles. Shine Lawyers are pursuing a class action against manufacturer American Medical Systems after women allegedly suffered painful complications their products. | Australian Broadcasting Corporation |  |  |  |  |  |
|  | McCarthy J. | 2016 | Agony of years suffering in isolation | Sydney Morning Herald |  |  |  |  |  |
|  | Wordsworth M. | 2016 | Women left in chronic pain after having now-banned TFS device implanted | ABC Premium News |  |  |  |  |  |
|  | Bowden E, McCarthy J. | 2017 | 'Nightmare' over: pelvic mesh set to be banned  [Published as “Complaints result in banning of pelvic mesh” in Sydney Morning Herald] | The Age |  |  |  |  |  |
|  | Miles J. | 2017 | WOMEN BREAK THE SILENCE. | The Courier - Mail. |  |  |  |  |  |
|  | Scott S, Branley A. | 2017 | Vaginal mesh implants: Class action against Johnson and Johnson begins in Federal Court | ABC Premium News |  |  |  |  |  |
|  | McCarthy J. | 2017 | Mum dies after pelvic mesh implant | The Sydney Morning Herald |  |  |  |  |  |
|  | Branley A. | 2017 | Vaginal mesh class action against Johnson and Johnson begins | Australian Broadcasting Corporation |  |  |  |  |  |
|  | Knaus C. | 2017 | Vaginal mesh risks downplayed by Johnson & Johnson, court told | The Guardian |  |  |  |  |  |
|  | Moodie C. | 2017 | Vaginal mesh implants: Gynaecologist urges proactive response to health concerns | ABC Premium News |  |  |  |  |  |
|  | Knaus C. | 2017 | Vaginal mesh lawsuit: executive denies prioritising Johnson & Johnson interests | The Guardian |  |  |  |  |  |
|  | Moore H, Rahman K. | 2017 | ‘I would not want my wife to undergo this procedure’: Johnson & Johnson doctor flagged concerns over ‘defective vaginal mesh’ - as company faces class action | Mail Online |  |  |  |  |  |
|  | McCarthy J. | 2017 | 'I would not want my wife to undergo this procedure': doctor | Sydney Morning Herald |  |  |  |  |  |
|  | Overington C. | 2017 | Crusade against mesh ‘a snow job’  [Published as “Doctors fight ‘crusade against vaginal mesh’” in The Australian – Online] | The Australian |  |  |  |  |  |
|  | Davey M. | 2017 | Therapeutic Goods Administration rejects claims it is 'too close' to medical industry | The Guardian |  |  |  |  |  |
|  | Knaus C. | 2017 | Johnson & Johnson vaginal mesh presentation featured lingerie-clad women, court told | The Guardian |  |  |  |  |  |
|  | Knaus C. | 2017 | Johnson & Johnson ?tried to prevent report about pelvic mesh devices, court hears | The Guardian |  |  |  |  |  |
|  | White N, Byrom R. | 2017 | 'The pain is like petrol burning inside me': Mother in constant 'unbearable' agony from a faulty medical implant slowly disintegrating inside her vagina | Mail Online |  |  |  |  |  |
|  | Scott S. | 2017 | Vaginal mesh implants: Johnson and Johnson defends product in court | ABC Premium News |  |  |  |  |  |
|  | Knaus C. | 2017 | Johnson & Johnson doubts vaginal mesh implants cause chronic pain, court hears | The Guardian |  |  |  |  |  |
|  | Scott S. | 2017 | Lamborghinis, ski trips used to market mesh implants to surgeons, documents show | Australian Broadcasting Corporation |  |  |  |  |  |
|  | Knaus C. | 2017 | Surgeons lacked caution in use of vaginal mesh implants, doctor admits | The Guardian |  |  |  |  |  |
|  | Alexander L. | 2017 | Mesh implant dangers | The Saturday Paper |  |  |  |  |  |
|  | McArthur G. | 2017 | Help for mesh pain | Herald Sun |  |  |  |  |  |
|  | McCarthy J. | 2017 | Mother's surgery nightmare: PELVIC MESH SCANDAL | Sydney Morning Herald |  |  |  |  |  |
|  | McCarthy J. | 2017 | ‘I can’t let the hurt be buried and forgotten’ | The Age. |  |  |  |  |  |
|  | O'Leary C. | 2017 | Battle to beat pelvic mesh pain goes east | The West Australian |  |  |  |  |  |
|  |  | 2017 | Vic Government steps up inquiry into implant safety | The Courier - Mail |  |  |  |  |  |
|  | Scott S. | 2017 | Controversial vaginal mesh implants banned because of risk to prolapse patients | ABC Premium News |  |  |  |  |  |
|  | Davey M. | 2017 | Australia bans transvaginal mesh products as 'too risky' | The Guardian |  |  |  |  |  |
|  | Darvall K. | 2017 | 'I could never have sex again': How a vaginal mesh implant left a Sydney woman in horrific pain and her husband too scared to touch her - as hundreds launch class action | Mail Online |  |  |  |  |  |
|  | Overington C. | 2017 | ‘Hysterical bias risks success of female surgery’ | The Australian |  |  |  |  |  |
|  | Mutton S. | 2018 | 'Not all vaginal implants are a problem': Doctor says women are refusing to seek treatment for incontinence after hundreds claimed mesh left them in pain and ruined their sex lives | Mail Online |  |  |  |  |  |
|  | Davey M. | 2018 | The biggest lesson from the vaginal mesh saga? Doctors must listen to women | The Guardian |  |  |  |  |  |
|  |  | 2018 | Treatment can improve quality of life | The Age |  |  |  |  |  |
|  | Knaus C. | 2018 | Johnson & Johnson accused of failing to warn patients at higher risk from vaginal mesh | The Guardian |  |  |  |  |  |
|  | Barr E. | 2018 | ‘What’s the price on our health?’ | Daily Telegraph​ - ​Online |  |  |  |  |  |
|  |  | 2018 | Clinic planned for women injured by mesh implants | The Courier - Mail |  |  |  |  |  |
|  | McCarthy J. | 2018 | Mesh victims demand ban after inquiry | The Sydney Morning Herald |  |  |  |  |  |
|  | Elliott E. | 2018 | SUFFERING IN SILENCE | Herald Sun |  |  |  |  |  |
|  | Davey M. | 2018 | Johnson & Johnson withdraws pelvic mesh device from Australian market | The Guardian |  |  |  |  |  |
|  | Moodie C. | 2018 | Pelvic mesh implant patients want answers from Senate report | ABC Premium News |  |  |  |  |  |
|  |  | 2018 | Researchers trial new pelvic prolapse treatment using stem cells, bioengineering | ABC Premium News |  |  |  |  |  |
|  | Withey A. | 2018 | Women living with prolapse - the horrific impact of traumatic birth and exercise | ABC Premium News |  |  |  |  |  |
|  | Knaus C. | 2018 | Transvaginal mesh inquiry criticises Australia's medical device regulation | The Guardian |  |  |  |  |  |
|  | Scott S. | 2018 | Pelvic mesh implants 'one of the biggest medical scandals' involving Australian women | ABC Premium News |  |  |  |  |  |
|  | Knaus C. | 2018 | Transvaginal mesh use should be suspended, health groups say | The Guardian |  |  |  |  |  |
|  | Scott S. | 2018 | Pelvic mesh implants: Further legal action on the table after scathing Senate report | ABC Premium News |  |  |  |  |  |
|  | Nigro L. | 2019 | New female implant class action uncovers decades of pain | Daily Telegraph​ - ​Online |  |  |  |  |  |
|  | Clun R, Cunningham M. | 2019 | Inquiry delays leave doctor free to practise  [Published as “Pelvic mesh trial investigation still under way after two years” in The Sydney Morning Herald – Online and as “Watchdogs delay pelvic mesh trial ethics probe” in The Age] | The Sydney Morning Herald |  |  |  |  |  |
|  | Hooton A, McCarthy J. | 2019 | The 'eight-minute' cure: how transvaginal mesh sentenced thousands of women to a life of pain | The Sydney Morning Herald - Online |  |  |  |  |  |
|  |  | 2019 | Pelvic mesh decision | Daily Telegraph​ - ​Online |  |  |  |  |  |
|  | Clun R. | 2019 | Australian goes overseas to run trial for risky procedure  [Published as “Australian doctor face of international clinical trial for controversial pelvic mesh” in The Sydney Morning Herald – Online and as “Doctor the face for new mesh trial” in The Age ] | The Sydney Morning Herald |  |  |  |  |  |
|  | Clun R. | 2019 | 'Nonsensical': planned pelvic mesh trial still registered despite former surgeon's comments | The Sydney Morning Herald - Online |  |  |  |  |  |
|  |  | 2019 | Women beat medical giant in pelvic mesh case | Daily Telegraph​ - ​Online |  |  |  |  |  |
|  | Knaus C. | 2019 | Australian women win landmark vaginal mesh class action against Johnson & Johnson | The Guardian |  |  |  |  |  |
|  | Clun R. | 2019 | Pelvic mesh trial still registered | The Sydney Morning Herald |  |  |  |  |  |
|  | Robinson N. | 2019 | Payout to women for mesh damage  [Published as “Pelvic mesh maker loses class action” in The Australian – Online] | The Australian |  |  |  |  |  |
|  | Walter A. | 2019 | ‘My post-baby bod cost me my marriage’ | newscomau |  |  |  |  |  |
|  | Costin L. | 2019 | Mesh mess victory | The Daily Telegraph |  |  |  |  |  |
|  | Miles J. | 2019 | Finally, an end to implant pain | The Courier - Mail |  |  |  |  |  |
|  | Casben L. | 2020 | Johnson & Johnson to pay $2.6m to women with faulty pelvic mesh implants | ABC Premium News |  |  |  |  |  |
|  | Gair K. | 2020 | Pelvic mesh maker to pay $2.6m in damages | The Australian - Online |  |  |  |  |  |
|  | Whitbourn M. | 2020 | Johnson & Johnson to pay $2.6 million for faulty pelvic mesh implants | The Sydney Morning Herald - Online |  |  |  |  |  |
|  | Gair K. | 2020 | $2.6m pelvic mesh payout | The Australian |  |  |  |  |  |
|  | Whitbourn M. | 2020 | Judge orders Johnson & Johnson to issue graphic warnings on pelvic mesh products  [Published as “Pelvic mesh products warning order” in The Age] | The Sydney Morning Herald - Online |  |  |  |  |  |
|  | Bonyhady N. | 2020 | Scrutiny to fall on class actions that vindicate victims  [Published as “Rebecca felt hopeless before she joined a class action” in The Sydney Morning Herald – Online] | Sydney Morning Herald |  |  |  |  |  |
|  | Butler B. | 2020 | Fears a crackdown on class actions in Australia could let big businesses 'do what they like' | The Guardian |  |  |  |  |  |
|  | Digirolamo R. | 2020 | Medical mesh mess widens | The Advertiser |  |  |  |  |  |
|  | Wells J. | 2021 | Johnson & Johnson loses pelvic mesh court appeal | ABC Premium News |  |  |  |  |  |
|  | Wells J. | 2021 | Johnson & Johnson loses pelvic mesh court appeal | MSN |  |  |  |  |  |
|  |  | 2021 | Johnson & Johnson loses pelvic mesh court appeal | Australian Broadcasting Corporation |  |  |  |  |  |
|  | Sainty L. | 2021 | Johnson & Johnson lose appeal over pelvic mesh devices that left women with debilitating pain and medical complications | Newscomau |  |  |  |  |  |
|  | Whitbourn M. | 2021 | Ruling paves way for Johnson & Johnson to pay millions for faulty implants | The Sydney Morning Herald - Online |  |  |  |  |  |
|  | Sainty L. | 2021 | Women’s health horror victory | The Advertiser |  |  |  |  |  |
|  | Sainty L. | 2021 | Women’s court win medical negligence | The Daily Telegraph |  |  |  |  |  |
|  | Whitbourn M. | 2021 | Court orders pharma giant to pay millions for faulty implants | The Sydney Morning Herald |  |  |  |  |  |

# Supplement 3 –Table – Examples of women’s illness narratives and women’s quotes

(*Article numbers are referring to the provided table in supplement 2)

| Examples | Article* |
| --- | --- |
| "The pain I have now I can do nothing about. Nothing I take or do takes it away," If you sit for too long, it's like the pressure is on the anchor and it's just pushing, pushing, pushing into the nerve until, if I am in the car I have to ask my husband to stop, I have to get out. "Standing for too long I feel like everything is going to fall out." …"They really can't take the anchors out. They are so embedded that you haemorrhage so badly no one has been able to do it," | 13 |
| "I didn't have any trouble before the operation," she returned to her surgeon three times after her operation to advise him of her pain but was told: "There's nothing wrong." | 15 |
| "This is insane. To almost be on your death bed at only 42 because of mesh. It's not right."  One month later, the Canadian mother of two was dead. | 17 |
| "I've literally been praying for nine years that this would come to light, that there would be accountability for what has happened to us,"  "For so many years we've been told there's nothing wrong with us, that symptoms are either in our head or it's not because of the mesh.  "I don't think people understand [there's not] one day from the time it's been put in that we haven't had pain - not one day." | 16, 29,39 |
| 'It's like sandpaper inside you that every now and then rears its ugly head and pokes through organs and walls, which affects nerves and how they function,'  'It's a terrible thing that rubs and creates a cheese grater effect.'  Even with full medical coverage, it still cost her about $20,000 to have the implant removed from one of the two doctors able to carry out the procedure, she said. | 22 |
| 'There was no choice, I was told this was the only option.'… 'I could never have sex again.'… 'He told me there was nothing wrong,' ... 'He said "I can examine you with two fingers so you're big enough (to have sex)".  'Just yesterday I was at hospital having injections all over my body to try and ease the pain,' the chronic pain started four years after the operation and worsened each year. She said the pain forced her to give up her 'dream job'. 'It totally changed my life.  'I've had so much bad health since that operation. There are days that I can't get out of bed and there are days that I try to do things and I just can't. | 22, 41 |
| “The raw pain of the erosion is like broken glass in your rectum and vagina. As we speak I am losing blood from my rectum and vagina and the pain is unbearable. This has totally destroyed my life,” …. By 14 months post-surgery the pain was unbearable and she began having transient ischaemic attacks, or 'mini strokes'. [She] cut her work hours to two days a week before her worsening condition forced her to quit altogether, and ruined her active fitness and social life. Now she can't even sit still long enough to watch a movie, pick up her grandchildren, or walk around without a cane. She has to sleep on her side in a hammock. | 28 |
| “On a bad day my son would have to take me to the toilet.... I feel like I have failed my kids, who are eight and 10. I feel like I have robbed them of their childhood. I feel like I have failed my partner” | 31 |
| she was told the mesh would make her feel like a “16-year-old virgin” and that she would be back at the gym in 10 days.  “To this day, I can’t sit upright on a chair for longer than 15 minutes at a time due to the searing, burning pain that travels across my lower abdomen and into my pelvis,” she said. I describe my pain as being cut open and set alight” “A deep burning, searing ache that intensifies with movement.” | 32 |
| “I woke in excruciating pain,…. I had pain like I have never experienced in my life and the staﬀ couldn’t get it under control….. After the surgery I had bruising from my groin down to my knees, with big puncture wounds in my buttocks….. I had four major surgeries for mesh erosion” | 33 |
| [Six weeks after initial TVM surgery] Her surgeon tried to remove the mesh…. She had further unsuccessful surgery to treat the complications…  In her final 16 months [she] could not urinate without a catheter. "She had to lay on the ground to use the catheter and she had to do that about every two hours"  …"She couldn't attend our father's funeral because there were no facilities at the crematorium to lay down and do what she had to do. Her life was reduced to places within two hours of her home, and only where she could lay down to catheterise herself  …took her own life, only two weeks after a doctor told her nothing could be done to ease her pain or address the devastating consequences of TVM device surgery | 35 |
| The 47-year-old is one of a steady stream of Australian women with pelvic mesh implants who have spent tens of thousands of dollars flying to a clinic in Missouri to have their implants removed.  "Every movement, you can feel the raw edge of the mesh inside you,"… she was using a walking stick to get around. Now, she is in a wheelchair.  She says she has tried to have her four implants removed but doctors will only agree to remove one of them. "It's indescribable, mesh pain. I'd [rather] have a baby any day, honestly.”  [She] was exhausted from years of chronic pain - and had just survived a suicide attempt - when she decided to cash in her life savings to pay for surgery in the United States.  "This has been a terrible betrayal of women," "Every person I consulted eventually put me onto a psychiatrist because they thought it was all in my head." | 52 |
| She said it ruined her life. "The Senate inquiry was a welcome step-forward for sufferers like me,"…. "The only reason any of us talk about the pain we've endured following our ordeal with this product is to impact change and prevent any other women from going through what we have," | 56 |
| She is 29 years old. Three years ago, she was a healthy mother of three boys living in a small town in Victoria. She worked as a dental assistant, and wanted to study nursing….. She can't work, or walk to the local park with her kids, or exercise her border collie; she needs a wheelchair for long outings…"My kids are paying the price for this," | 61 |
| Eventually I had a surgery to “trim” the mesh but that cut the tension and the mesh rolled up into my body and I became incontinent. The surgeon said this was so rare and he’d never seen it before. I went from being a 45-year-old mother of two, who worked four days a week, who loved to watch her kids play sports and volunteered at the school, to being in chronic pain. It was devastating and it only got worse. I had mesh protruding back through my vagina, in two spots and the doctors said they couldn’t do anything for me. I was the “only one”. I’d be curled up in bed for hours or days at a time, with pain, tiredness, brain fog, it was hell. I became unreliable as a friend, as a wife, as an employee. My kids would come in and lay with me or talk to me in bed. My relationship became very fractured during this time, and eventually ended. | 69 |
| It's affected our womanhood, our relationships with our partners, our time with our children &our families ... (the ability) for some people to work- People just don't understand how much it impacts your life | 72 |
| “It’s horrible,….It’s embarrassing. I just want to be normal and be able to go out and not urinate all over myself.” | 79 |
| she could no longer have sex. She has back and leg pain and "shooting electrical shock pain from my lower body up through to my head"….she had suffered "permanent incontinence" | 36 |
